# Effects of collagen peptides from *Micropterus salmoides* skin on oxidative damage induced by cyclophosphamide in mice

**DOI:** 10.3389/fnut.2022.1037212

**Published:** 2022-11-03

**Authors:** Mengyao Han, Zhongshan Zhang, Xinyue Li, Haibin Tong, Zhiguo Xu, Zikang Ding, Anquan Yang, Min Xie, Xiaomei Wang

**Affiliations:** ^1^Zhejiang Provincial Key Laboratory of Aquatic Resources Conservation and Development, Huzhou University, Huzhou, China; ^2^College of Life and Environmental Sciences, Wenzhou University, Wenzhou, China; ^3^School of Life and Health, Huzhou College, Huzhou, China; ^4^Osmum Biological Co., Ltd., Deqing, China

**Keywords:** *Micropterus salmoides*, collagen peptides, cyclophosphamide, oxidative stress, gut microbiota

## Abstract

To investigate the protective effect of collagen peptide from *Micropterus salmoides* skin (CPMs) on oxidative damage induced by cyclophosphamide in mice. Balb/c female mice were divided into blank, model (cyclophosphamide, CTX), positive control (levamisole hydrochloride), and collagen peptide low-, medium-, and high-dose groups. The results showed that CPMs increase the body mass and immune-related organ indexes, such as liver and kidneys of immunosuppressed mice. The activities of ALT, AST, UA, BUN, and MDA in the liver and kidney tissues decreased significantly, while those of SOD and GSH-Px increased significantly. CPMs can relieve the pathological damage to immune organs. CPMs significantly increase the activities of IL-2, IgG, and TNF-α in serum and SOD activity, while the MDA content was decreased compared to the model group. CPMs can exert a protective effect on cyclophosphamide-induced oxidative damage and have application prospects in the field of health food.

## Introduction

Cyclophosphamide (CTX) is an alkylating anticancer agent that causes myelosuppression, immunosuppression, oxidative stress, and other side effects and is widely used to treat various cancers and diseases, such as systemic lupus erythematosus, lymphoma, and autoimmune diseases (1, 2). However, killing tumor cells also damages healthy cells in addition to a strong immunosuppressive effect; therefore, CTX is used as an immunosuppressive drug in animal experiments. Previous studies have shown that CTX has significant hepatic and renal toxicity and is activated by the cytochrome P450 oxidase system of hepatic microsomal enzymes to produce two metabolites, phosphoramidite nitrogen mustard and acrolein, and the byproduct chloroacetaldehyde, which also has marked hepatic and renal toxicity (3, 4). Ovaries are the main reproductive organs in women. CTX can cause symptoms, such as irregular menstruation, amenorrhea, infertility, and premature ovarian failure, in female tumor patients, which seriously affects the physical and mental health of female patients (5).

Collagen is the main component of connective tissue and a high molecular weight protein with white and opaque characteristics. It is widely distributed in animals, mainly in skin and ligaments, and is also the most abundant protein in fish skin and scales, supporting the organs and protecting the body (6). The proportion of fish skin to the total weight of fish varies with different types of fish. Taking the proportion of fish skin to the total weight of fish as 5∼15%, the annual output of fish skin was about 2 million tons. These fish skin are discarded in large quantities during processing, causing serious resource waste and environmental pollution. Extracting collagen from fish skin can not only improve the added value of fish products, but also reduce the waste of fish skin and the resulting environmental pollution (7–10). Collagen peptides are enzymatic products of collagen, consisting of 3–20 amino acids (11, 12) and possessing antioxidant, lipid-lowering, immunity-boosting, and other biological activities.

The cellular and humoral immune pathways of the immunomodulatory effects of collagen peptides from *Micropterus salmoides* skin (CPMs) have not been studied in depth. Therefore, the present study reported the effects of CPMs on CTX-induced immunocompromised mice and explored the underlying mechanism of action.

## Materials and methods

### Samples

*Micropterus salmoides* was obtained from Zhejiang Provincial Key Laboratory of Aquatic Resources Conservation and Development. Female BALB/c mice (18–22 g) were purchased from Hangzhou Ziyuan Experimental Animal Technology Co., Ltd. (SCXK20190004). CTX and levamisole hydrochloride were purchased from Aladdin Reagent Co. (Shanghai, China). Mouse TNF-α, IL-2, and IgG ELISA, superoxide dismutase (SOD), malondialdehyde (MDA), and glutathione peroxidase (GSH-Px) kits were purchased from Nanjing Jiancheng Bioengineering Institute (Nanjing, China).

### Preparation of collagen peptides

The collagen peptides were prepared, as described previously (13). After degreasing, the collagen of the skin was extracted using the acid enzyme method. Alkaline protease was added to the above collagen solution to obtain alkaline protease-hydrolyzed collagen peptides (CPMs).

### Animal feeding and grouping

The mice were housed in a barrier environment at 24 ± 0.5°C, 50–60% humidity, and 12 h alternation between day and night. Subsequently, the animals were randomly divided into six groups, 10 in each group. Except for the normal control group (CK), which was given saline solution once/day, the other five groups were administered CTX (0.2 ml) 80 mg/kg body weight/day by intraperitoneal injection for 7 days. From days 8–28, the model control group was gavaged with saline 0.2 ml, the collagen peptide low-, medium-, and high-dose groups were gavaged with 0.2 ml collagen (50, 100, and 200 mg/kg body weight/day), and the positive control group was administered levamisole hydrochloride 0.2 ml (40 mg/kg body weight/day) by gavage. The animals were fed and watered freely during the experiment. After the last gavage, the animals were fasted for 24 h and executed by blood collection from the eyes.

### Histopathological analysis of liver and kidney

After 21 days, the body weight was measured, and the mice in each group were executed by blood collection from the eye. The liver and kidneys were removed and weighed. The organ index was calculated as follows:


$$\text{Organ index (mg per 10 g)}=\frac{\text{weight of liver or kidneys (mg)}}{\text{body weight (g)}}$$

The liver and kidney were fixed in 4% (v/v) formaldehyde solution, embedded in paraffin, and stained with hematoxylin-eosin (HE staining) to observe and photograph the histomorphological changes of the thymus and spleen under the microscope.

### Enzyme activity and cytokine analysis

Liver and kidney tissues were homogenized in 0.9% saline to make a 10% homogenate. The supernatant was obtained by centrifugation of the homogenate at 2,500 rpm for 10 min, and the activity of ALT, AST, BUN, UA, and antioxidant enzymes were measured (14). The serum was obtained by centrifugation (1,500 *g*, 5 min) of the blood collected from mouse eyes, and the level of TNF-α, IL2, and IgG was determined using the ELISA kits (15).

### 16S rRNA sequencing of gut microbes

High-throughput sequencing was used to study the effect of collagen peptides from the skin of largemouth bass on the intestinal flora of mice. Fresh mouse feces were collected in sterilized centrifuge tubes and stored at −80°C. Subsequently, the fecal samples were shipped on dry ice to Shenzhen Microcomputer Technology Group for sequencing. Genomic DNA was extracted from mouse fecal samples by the CTAB method and used as a template in sterile water at 1 ng/μl for PCR amplification. The amplified products were mixed with 2% agarose gel at equal concentrations. A volume of 10 μl of the upper sample was aspirated, and 1× TAE buffer was added to start electrophoresis to purify and recover the PCR products. The library was constructed using the TruSeq kit, quantified, tested, and sequenced on the machine.

### LC-MS non-targeted metabolic assay

Stool samples were ground in liquid nitrogen, weighed 100 mg into a 1.5-ml centrifuge tube, 500 μl of 80% methanol in water added, mixed well, and placed in an ice water-bath at 4°C for 5 min. Then, the supernatant was collected and analyzed by LC-MS (16). The data file was imported into the software to pick each metabolite. After the selection, the sample was aligned to the peaks in the software. These peaks were extracted, and the peak areas were quantified. The software was also used to infer the molecular formulae based on the ion peaks and fragment ions and placed in a database for comparison. The raw quantification results were normalized to identify the metabolites and obtain relative quantification results.

### Statistical analysis

The data were expressed as mean ± standard deviation (SD) (*n* = 6). Differences between the means of experimental and control groups were analyzed statistically using Duncan’s multiple range test, represented by alphabets for each level of significance. *P* < 0.05 indicated a statistically significant difference.

## Results

### Characteristics of collagen peptides *Micropterus salmoides* skin

According to SDS-PAGE, the collagen from *M. salmoides* skin was mainly composed of two 120 kDa α Chain and a 200 kDa β Chain, consistent with the characteristics of type I collagen. The maximum absorption peak of collagen was at 234 nm. The absorption peaks of collagen can be seen from the infrared spectrum, mainly including amide A, B, I, II, and III bands, which is consistent with the previous report (17). The small molecular peptides produced by the enzymatic hydrolysis of proteins are easily absorbed by the human body. Collagen can be solubilized in the enzymatic hydrolysis solution in the form of a polypeptide or amino acid after hydrolysis for 4 h. Excessive hydrolysis may reduce or completely lose the functional properties and biological activity of collagen (18). The degree of hydrolysis of alkaline protease can reach 25.44%. According to the mass spectrum, the molecular weight of CPMs was concentrated in 110–1,942. CPMs contain 12 amino acids, with a high content of glycine, alanine, glutamate, and hydroxyproline. The antioxidant capacity is closely related to the type, number, and sequence of amino acids. Previous studies have shown that acidic amino acids (Asp and Glu), aromatic amino acids (Phe, Tyr, and Trp), sulfur-containing amino acids (Met and Cys), and hydrophobic amino acids have a strong antioxidant capacity (19).

### Effect of collagen peptides *Micropterus salmoides* skin on body weight and organ indices

As seen in Table 1, the initial body weights of the mice in each group did not differ significantly, in accordance with the principle of random assignment. At the end of the gavage, the final body weight of the PC group increased significantly (*P* < 0.05) compared to the M group. CTX increased the liver and kidney indices, indicating CTX-induced enlargement of the liver and kidneys, while the CPM group showed an improvement in the recovery of the liver and kidney index.

**TABLE 1:** Effect of samples on liver, kidney, and ovarian organ indices in mice.

Group	Initial weight/g	Final weight/g	Liver index/(mg.g^–1^)	Kidney index/(mg.g^–1^)
CK	21.05 ± 0.77	19.49 ± 0.79	46.42 ± 2.57	13.26 ± 0.89
M	21.16 ± 1.04	18.63 ± 1.24	49.36 ± 3.30	14.01 ± 0.54
PC	21.60 ± 1.30	20.32 ± 1.08*	44.23 ± 2.23**	13.92 ± 1.05
CL	21.16 ± 0.47	19.98 ± 0.91	45.18 ± 3.02	14.18 ± 1.46
CM	21.19 ± 1.02	19.10 ± 0.58	46.84 ± 3.35	14.13 ± 0.51
CH	21.14 ± 1.21	18.75 ± 0.85	44.77 ± 3.85	13.75 ± 0.95

Compared with the M group, * indicates *P* < 0.05, ** indicates *P* < 0.01; compared with the CK group.

### Histopathological analysis of liver and kidney

**FIGURE 1:**
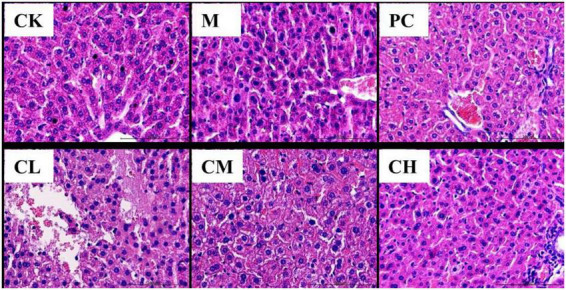
Histopathological changes in the liver (HE, 200×).

As shown in Figure 1, the liver of mice in the CK group was intact, with uniformly distributed hepatic cords and uniformly colored and arranged hepatocytes. It had a normal hepatic sinusoidal structure and a large number of binucleated hepatocytes. The liver of mice in the M group had obvious lesions, with a significant decrease in the number of binucleated hepatocytes. They have edema of hepatocytes, blurred and illegible hepatic cords, dilated and bleeding hepatic sinusoids, and erythrocytes and neutrophils in the sinusoids. The hepatocytes in the CL group were similar to those in the M group, with severe damage to the hepatocytes, loss of nuclear membrane, rupture of nucleus pulposus, and massive infiltration of erythrocytes and neutrophils in the portal canal and hepatic cords. In the CH and PC groups, a small number of hepatocytes were swollen, and the hepatic cords were visible.

**FIGURE 2:**
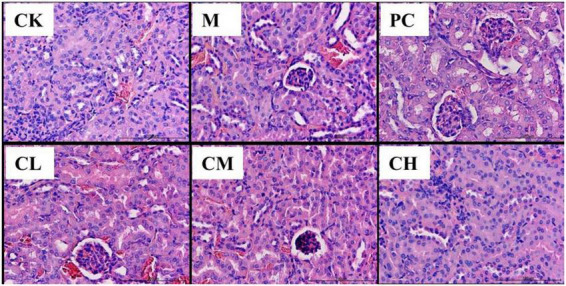
Histopathological changes in the kidney (HE, 200×).

The pathological sections of the kidneys of the mice in the CK group are shown in Figure 2. The kidneys of the mice in the M group showed pathological changes, such as a small interstitial space, a large number of blood cells in the interstitium, and incomplete endothelial cells in the capsule wall. In the CM, CH, and PC groups, the degree of injury was significantly reduced, the kidney structure was intact, the renal capsule was visible, and a few blood cells were detected in the capsule.

### Effect of collagen peptides *Micropterus salmoides skin* on enzyme activity

**FIGURE 3:**
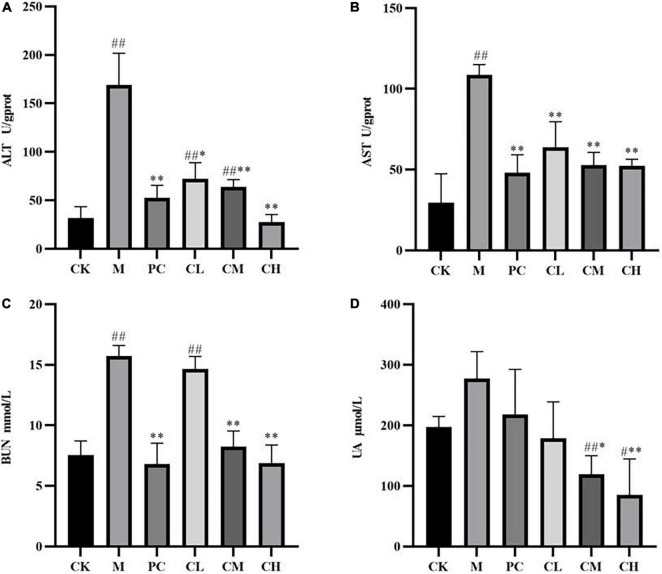
Effect of samples on liver ALT **(A)**, AST **(B)**, and renal BUN **(C)**, UA **(D)** activity in mice. (Compared with the M group, * indicates *P* < 0.05, ** indicates *P* < 0.01; compared with the CK group, ^#^ indicates *P* < 0.05, ^##^ indicates *P* < 0.01).

Figure 3 shows the effects of collagen peptides on liver glutathione (ALT) and glutathione transaminase (AST) activities in CTX liver-injured mice. Compared to the CK group, the ALT and AST activities were significantly higher in the M group (*P* < 0.01), indicating that the mouse liver injury model was established successfully using CTX. After gavage of collagen peptide, the ALT and AST activities were significantly decreased in all the dose groups than in the M group (*P* < 0.05), and the effect of collagen peptide dose groups was similar to that of levamisole hydrochloride, and a high dose of collagen peptide significantly reduced the increase in ALT activity (*P* < 0.01).

Urea (BUN) and uric acid (UA) are major indicators of renal function, representing the degree of renal impairment, and can be used to assess the protective effect of peptides on the kidney. As shown in Figure 3, BUN increased significantly (*P* < 0.01), while the UA level increased but not significantly (*P* < 0.01) compared to the blank group after modeling. Also, no significant difference was detected between the CL and M groups, while the BUN and UA levels decreased significantly (*P* < 0.01) in the CM and CH groups. The results showed that collagen peptide had no significant effect on the BUN and UA levels in the low-dose group and reduced the CTX-induced kidney damage in the medium- and high-dose groups.

Compared to the CK group, the SOD and GSH-Px levels were significantly lower in the M group (*P* < 0.05), and MDA levels were higher in the CK group after CTX injection, indicating successful modeling. After the gavage of collagen peptide, the SOD and GSH-Px enzyme activities increased gradually and cleared the hepatic reactive oxygen species, while MDA levels decreased sequentially, suggesting a dose correlation. The results showed that collagen peptides effectively scavenge free radicals in the body and slow down the oxidative process, thereby lessening the harm caused by immune depression.

**FIGURE 4:**
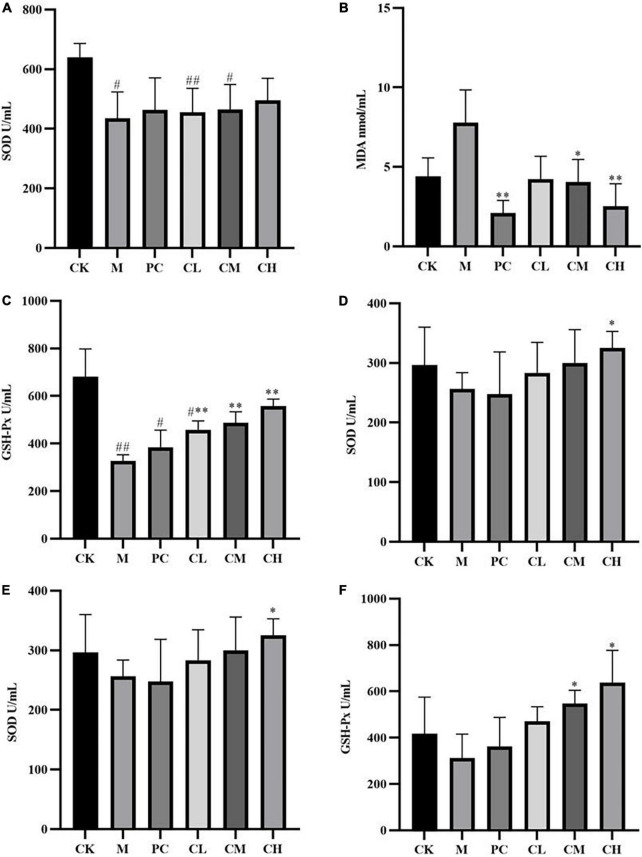
Effects of samples on the levels of SOD **(A)**, MDA **(B)**, and GSH-Px **(C)** in liver tissues and SOD **(D)**, MDA **(E)**, and GSH-Px **(F)** in kidney tissues. (Compared with the M group, * indicates *P* < 0.05, ** indicates *P* < 0.01; compared with the CK group, ^#^ indicates *P* < 0.05, ^##^ indicates *P* < 0.01).

Kidney is a crucial excretory organ that regulates the immune function of the body and maintains the homeostasis of the body’s internal environment (20). Free radicals can disrupt the oxidative balance of the body, causing damage to the liver and kidneys and resulting in immune deficiency and damage to the body (21). Figure 4 shows that SOD and GSH-Px enzyme activities are significantly higher in the CH group than in the M group (*P* < 0.05), which maintains the oxidative balance of the body, and the MDA level was significantly lower (*P* < 0.01). These results indicated that the high dose of collagen peptide increases the renal SOD and GSH-Px activities, decreases the MDA activity, reduces cell damage, and enhances the antioxidant capacity of mice.

### Effect of collagen peptides *Micropterus salmoides* skin on cytokines

**FIGURE 5:**
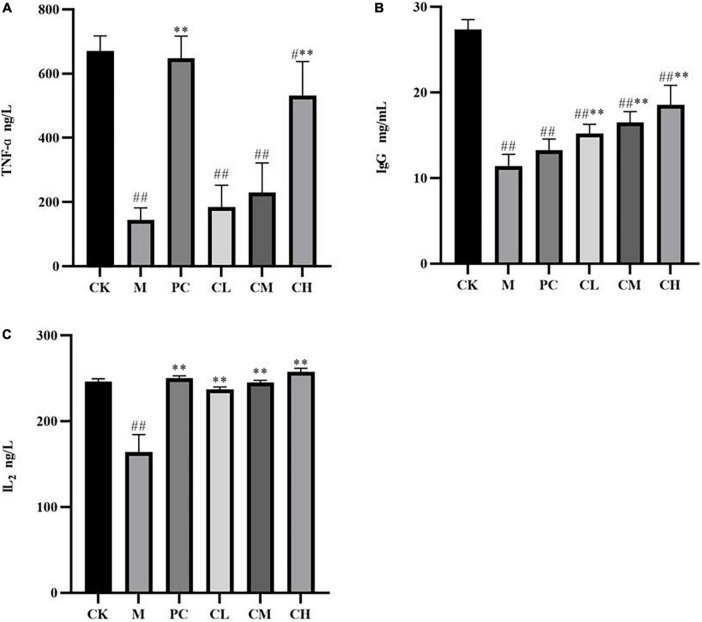
Effect of samples on serum TNF-α **(A)**, IgG **(B)**, and IL2 **(C)** levels in mice (Compared with the M group, * indicates *P* < 0.05, ** indicates *P* < 0.01; compared with the CK group, ^#^ indicates *P* < 0.05, ^##^ indicates *P* < 0.01).

After intraperitoneal injection of CTX, the levels of TNF-α, IL-2, and IgG decreased significantly (*P* < 0.01), and after gavage with collagen peptides, the cytokine levels increased gradually, with no significant difference in the TNF-α and IL-2 levels in the CH group compared to the CK group (*P* > 0.05), indicating that the immunity levels in the CH group returned to normal values (Figure 5). The higher the concentration of collagen peptide in the CH group, the more significant the difference was compared to the M group (*P* < 0.01), and the high-dose group and levamisole hydrochloride improved the immune function of mice and alleviated the effect of CTX on the organism.

### Analysis of the composition of intestinal flora

**FIGURE 6:**
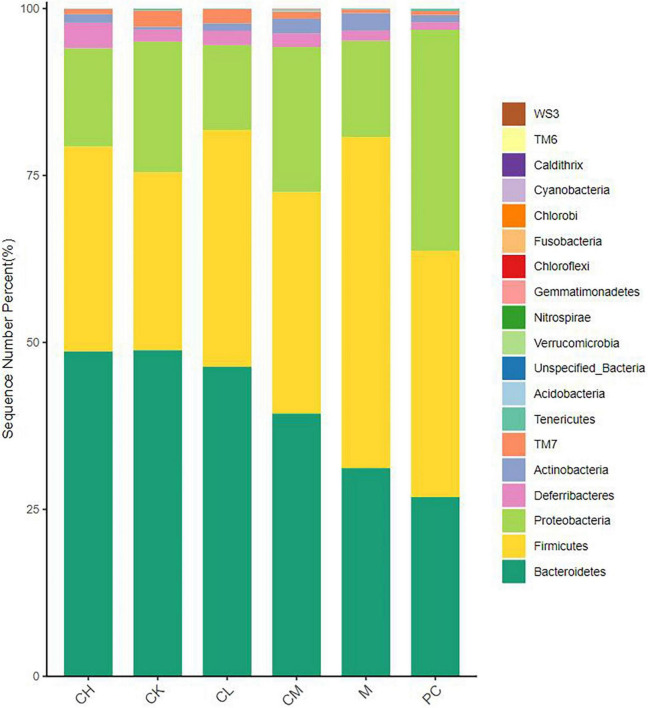
Relative distribution of gut microbiota at the phylum level (top 20 species in relative abundance).

As seen in Figure 6, the intestinal flora of mice in all groups mainly consisted of *Bacteroidetes*, *Proteobacteria*, and *Firmicutes*, which reached >90% of the total number of colonies. Compared to the M group, the collagen peptide dose group significantly increased the abundance of *Bacteroidetes* (*P* < 0.05) and decreased the abundance of *Firmicutes*. This finding indicated that collagen peptide improves the CTX-induced disorder of intestinal flora, helps restore the intestinal microecological balance, and improves immune function in mice.

### LEfSe analysis of intestinal flora species

**FIGURE 7:**
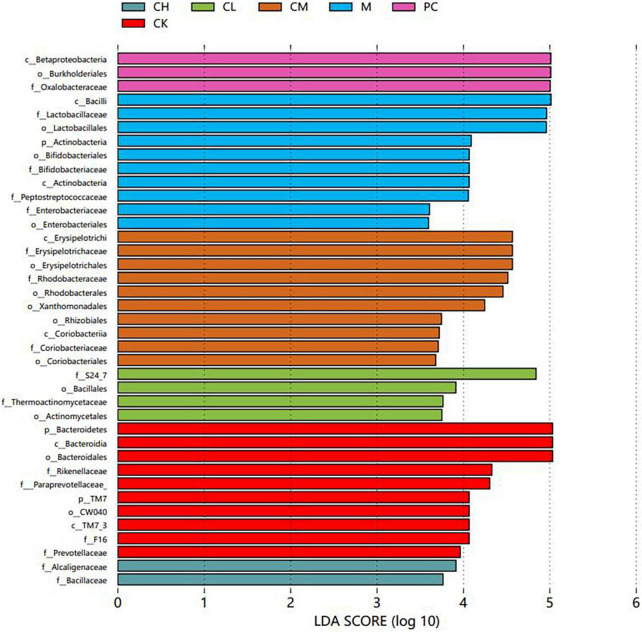
LDA histogram of gut microbiota in mice.

**FIGURE 8:**
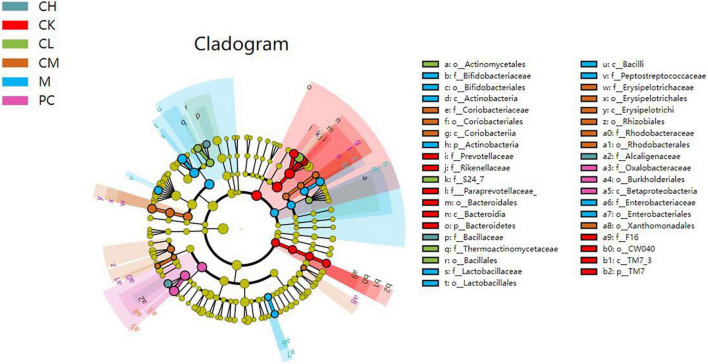
Cladogram graph of gut microbiota in mice.

The linear discriminant analysis effect size (LEfSe) method is based on a relative abundance table that combines non-parametric tests and linear discriminant analysis to evaluate the differences in population abundance. LEfSe identifies the characteristic microorganisms (microorganisms with latent dirichlet allocation (LDA) > threshold) within each group, and the taxonomic hierarchy of the characteristic microorganisms is shown in Figure 7. The main taxa with the above differences are represented in the figure by class intervals and names, while the categories below the family are only color-coded, indicating significant differences in the microbial evolutionary correlations among the groups. In the LDA bar graph, the horizontal coordinate represents the LDA value; the higher the value, the greater the difference. The vertical coordinate represents the species, and the color of the bar corresponds to the group; the characteristic microorganisms show a high abundance ratio within the group. The cladogram graph corresponds to the classification levels of kingdom; phylum, order, family, and genus from inside to outside and the lines between the levels represent the affiliation (Figure 8). Each circle node represents a species; yellow nodes represent non-significant differences between groups, and colored nodes represent the characteristic microorganisms of the corresponding group. The colored sector area shows the range of subordinate categories of the characteristic microorganisms.

Compared to the M and dose groups, the CK group contained abundant *Prevotellaceae*, *Bacteroidetes*, and *Rikenellaceae* in the intestine. After CTX injection, the difference between the M and the other groups was detected in the presence of *Bacteriaceae*, *Bifidobacteriaceae*, *Lactobacillus*, *Actinobacteriaceae*, and *Enterobacteriaceae*. The intestine of mice in the dose group harbored *Bacillus*, *Enterobacteriaceae*, and *Lactobacillus*.

### Principal component analysis (PCA) of differential metabolites

**FIGURE 9:**
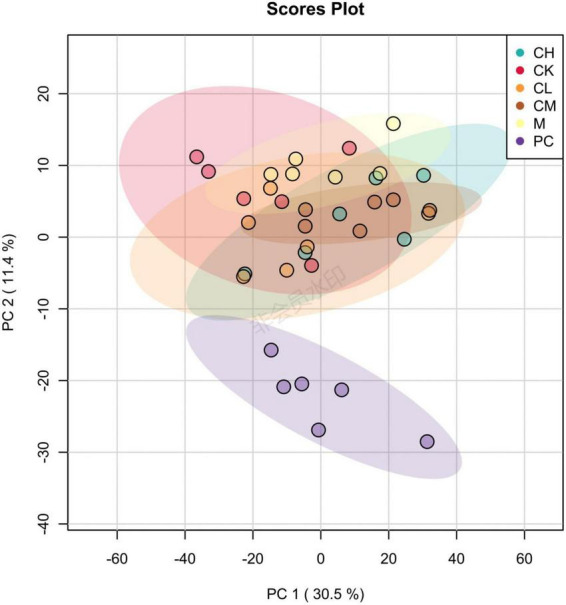
Principal component analysis (PCA) diagram of gut microbiota in mice.

The purpose of PCA is to select factors that do not interfere with each other, are representative of all metabolites, and preserve the maximum variance of the initial metabolites. As shown in Figure 9, the distinctly distributed point clouds in different areas indicate significant differences in the metabolite composition between the two groups. Interestingly, the differences in metabolite composition between the collagen peptide dose groups were small, while those between levamisole hydrochloride and the other groups were large.

### Metabolite content statistics

**FIGURE 10:**
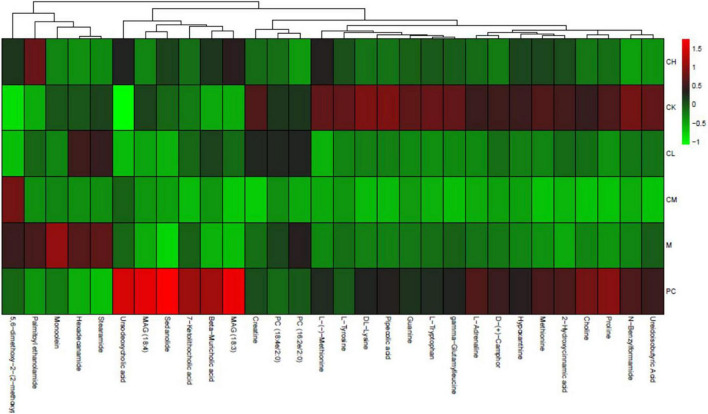
Metabolite heat map clustering results.

**FIGURE 11:**
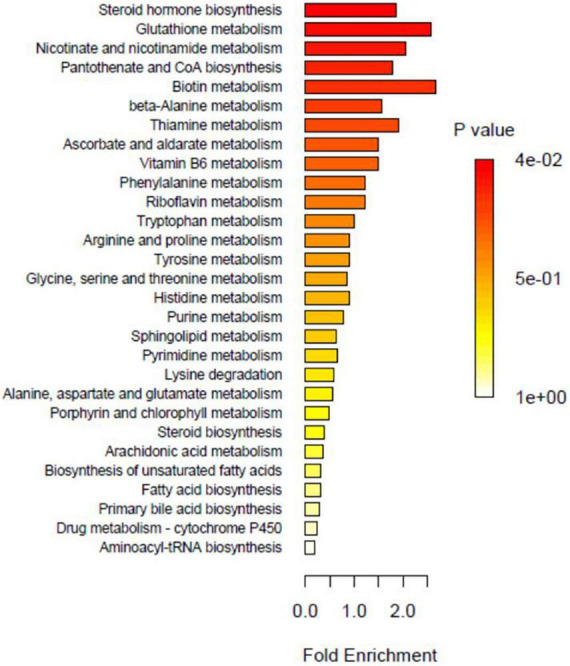
Over representation analysis (ORA) enrichment analysis.

The percentage of each metabolite was calculated for all samples and visualized in a stacked bar chart for the comparison of structural changes in the metabolite composition between the groups. Figure 10 shows the top 20 metabolites, with the remaining metabolites included in Others. The major metabolites include choline, methionine, stearamide, L-tyrosine, hexadecanamide, ursodeoxycholic acid, β-rhamnocholic acid, L-tryptophan, L-adrenaline, and 7-ketolithocholic acid. Among all metabolites, hormones and vitamins play a specific role in the organism. All metabolites were annotated using the Kyoto encyclopedia of genes and genomes (KEGG) database to deduce the biological role played by the metabolite, and the percentage content of each biological role was calculated and plotted in a stacked bar chart. As shown in Figure 10, the main groups include vitamins and cofactors, nucleic acids, steroids, hormones and transmitters, and peptides, which show the highest peptide content in the CK group; the peptide content decreases and the steroid content increases after CTX injection. The results of metabolite heat map clustering for each group are shown in Figure 11, where each row represents a sample, and each column represents a different metabolite, for the six groups of samples. Different colors represent the levels: red indicates upward adjustment, green indicates downward adjustment, and green to red indicates low to high metabolite levels. As shown in Figure 10, when the samples of different groups are distributed in various positions, the structure of the 30 metabolites varies greatly among the groups, and the metabolite composition was more different among the four groups of CK, M, PC, and collagen peptide dose group.

### Metabolic pathway analysis

Enrichment analysis enables the identification of biological pathways that play a decisive role in the biological processes and the analysis of the underlying molecular mechanisms. Before performing enrichment analysis, the differential metabolites must be screened and observed for significant enrichment in metabolic pathways. The pathways consisting of differential metabolites are significantly enriched, as shown in Figure 11. The major metabolic pathways include steroid hormone biosynthesis, nicotinate, and nicotinamide metabolism, glutathione metabolism, biotin metabolism, alanine metabolism, ascorbate and aldehyde metabolism, thiamine metabolism, vitamin B6 metabolism, pantothenate, and coenzyme A biosynthesis.

Based on the metabolic pathway map, the topology of metabolites is visible, and the upstream and downstream correlations and modes of action can be understood, which assist in identifying the genes associated with metabolites. Figure 11 shows the metabolic pathways that contain only metabolites. The metabolites indicated in red are of major concern and differ significantly between groups. Figure 11 shows the metabolic pathways that contain both metabolites and genes. Metabolites marked in red differ significantly between groups, with the color corresponding to the group relative to other groups, indicating higher metabolite levels in the corresponding group. Moreover, oxidized glutathione metabolism, coenzyme II metabolism, pyroglutamate metabolism, 2,5-diaminopentanoic acid metabolism, and spermidine metabolism were the metabolic pathways that differed significantly between groups.

## Discussion

Cyclophosphamide is a widely used anticancer drug, but it can damage healthy cells during chemotherapy and has a strong immunosuppressive effect. Cyclophosphamide is activated by the cytochrome P450 oxidase system of hepatic microsomal enzymes to produce two metabolites, phosphoramidite mustard and acrolein, and the byproduct chloroacetaldehyde (22). Phosphoramidite mustard kills cancer cells through apoptosis, acrolein is toxic to normal cells as it induces apoptosis and necrotic cell death, and chloroacetaldehyde exerts significant nephrotoxicity (23, 24). Some studies have shown that bioactive peptides improve CTX-induced immunosuppression (25). CTX-induced toxicity in the liver and kidneys elevates the levels of ALT, AST, BUN, and UA (3, 26); Bhat et al. (27) administered XCTX *via* intraperitoneal injections in mice and observed that the liver was also infiltrated with inflammatory cells and hepatic blood sinusoidal congestion. Our results showed that CTX had no significant effect on the body weight but caused liver and kidney damage, resulting in elevated AL, AST, BUN, and UA levels. Conversely, collagen peptides ameliorated the effects of cyclophosphamide on mice.

Biological oxidation is an essential process in the living body, during which free radicals are produced; however, excessive radicals cause oxidative stress and are key factors in aging and diseases, such as Alzheimer’s disease, cardiovascular disease, and cancer (28, 29). Therefore, it is necessary to consume the antioxidants to remove the excess free radicals from the body, maintain the body’s redox balance, and thus prevent the occurrence of diseases. SOD and GSH-Px scavenge the reactive oxygen species from the body, maintain the stability of the internal environment of the organism, and are associated with the development of several diseases of the organism, such as cellular damage and aging (21). MDA is extremely biotoxic, impairs cellular functions, and causes damage to the organisms (30). CTX-induced liver and kidney toxicity are associated with oxidative stress caused by a decrease in antioxidant enzymes (31). In the present study, low levels of SOD and GSH-Px were detected in the liver and kidney of group M mice. However, the activity of these antioxidants was upregulated after the gavage of collagen peptides from the skin of *M. salmoides*. A significant increase was observed in the liver and kidney MDA levels in the mice injected with CTX. However, after the gavage of collagen peptides, MDA levels were significantly reduced.

Several studies have confirmed that CTX causes immunosuppression and reduces the immunity of the body (15). Immunogens stimulate cells to produce cytokines, which are small-molecule proteins that bind to the corresponding receptors to regulate cell growth, tissue repair, and specific and non-specific immune responses (32). Immunoglobulins play a major role in humoral immunity. IgG accounts for 80% of total serum antibodies and is widely distributed in the body, has antibacterial and antiviral effects, and has a critical immune function (33). TNF-α is a pro-inflammatory factor and is secreted in response to cellular infection, inflammation, and environmental stress. Moreover, collagen peptides can increase the level of immune factors, thus improving the immunity of mice.

Intriguingly, the intestine is the main source of nutrient intake and plays a major role in the body’s immunity. It intakes nutrients and maintains the growth of the body through digestion and absorption. On the other hand, it prevents harmful substances such as pathogenic bacteria from entering the body and protects the body from invasion by foreign pathogens (34). The intestine is the largest reservoir of microorganisms in the body and plays a role in immune regulation, metabolism, and maintenance of micro-ecosystem stability (35). It is widely believed that damage to the intestinal barrier by chemotherapy drugs leads to intestinal and systemic inflammation, diarrhea, enteritis, and systemic immunodeficiency, resulting in high mortality (36). Intestinal flora plays a crucial role in maintaining a healthy gut. Probiotics are in a dynamic balance with opportunistic pathogenic bacteria, and a disruption in this balance prompts infections in the organism (37). Compared to the CK group, the abundance of *Bacteroidetes* is decreased, and that of *Firmicutes* is increased in the M group, and the increase in the ratio of *Firmicutes*/*Bacteroidetes* was consistent with the results of Tian et al. (38); Huang et al. (39) used CTX to establish a mouse model of immunosuppression in mice and detected *Rikenellaceae*, *Streptococcaceae*, *Lactobacillaceae*, *Helicobacteraceae*, *Enterobacteriaceae*, *Bacteriaceae*, and *Bacillus* in the colon of the animals. The relative abundance of *Rikenellaceae*, *Streptococcaceae*, *Lactobacillaceae*, *Helicobacteraceae*, *Enterobacteriaceae*, and *Bacteroidaceae* increased but that of *Muribaculaceae*, *Saccharimonadaceae*, and *Peptococcaceae* decreased, which was similar to the current results.

Shuai et al. (40) demonstrated that the metabolic pathways significantly affected by CTX include arachidonic acid, pyrimidine, and glycerophospholipid metabolism. Compared to the M group, collagen peptides mainly affected amino acid metabolism, purine metabolism, and other pathways, regulated the content of amino acids and fatty acids in the intestine of mice and reversed the abnormal intestinal metabolic function to normal.

## Conclusion

*M. salmoides* skin collagen peptide improves the immunosuppression induced by CTX, improves the immunity of the body, and enhances the repair of liver and kidney, as well as ovarian function damage. It also regulates the intestinal flora of mice and affects intestinal metabolic function, thereby improving the immunity of mice.

## Data availability statement

The original contributions presented in this study are publicly available. This data can be found here: https://www.ncbi.nlm.nih.gov/sra/?term=PRJNA838136.

## Ethics statement

The animal study was reviewed and approved by Ethics Committee of Huzhou University, China.

## Author contributions

MH and ZZ: writing – original draft preparation. XL: conceptualization. HT: validation. ZD: data curation. AY and ZX: resources. MX: investigation. XW: writing – review and editing. All authors have read and agreed to the published version of the manuscript.
